# Optimizing the widely used nuclear protein‐coding gene primers in beetle phylogenies and their application in the genus *Sasajiscymnus* Vandenberg (Coleoptera: Coccinellidae)

**DOI:** 10.1002/ece3.6497

**Published:** 2020-06-28

**Authors:** Weidong Huang, Xiufeng Xie, Feng Peng, Xinyue Liang, Xingmin Wang, Xiaosheng Chen

**Affiliations:** ^1^ Guangdong Key Laboratory for Innovative Development and Utilization of Forest Plant Germplasm Department of Forest Protection College of Forestry and Landscape Architecture South China Agricultural University Guangzhou China; ^2^ Key Laboratory of Bio‐Pesticide Innovation and Application, Guangdong Province Engineering Research Center of Biocontrol Ministry of Education and Guangdong Province Guangzhou China; ^3^ Guangdong Agriculture Industry Business Polytechnic College Guangzhou China

**Keywords:** coccinellidae, nuclear protein‐coding gene, phylogenetic informativeness, phylogeny, *Sasajisycmnus*

## Abstract

Advances in genomic biology and the increasing availability of genomic resources allow developing hundreds of nuclear protein‐coding (NPC) markers, which can be used in phylogenetic research. However, for low taxonomic levels, it may be more practical to select a handful of suitable molecular loci for phylogenetic inference. Unfortunately, the presence of degenerate primers of NPC markers can be a major impediment, as the amplification success rate is low and they tend to amplify nontargeted regions. In this study, we optimized five NPC fragments widely used in beetle phylogenetics (i.e., two parts of carbamoyl‐phosphate synthetase: CADXM and CADMC, Topoisomerase, Wingless and Pepck) by reducing the degenerate site of primers and the length of target genes slightly. These five NPC fragments and 6 other molecular loci were amplified to test the monophyly of the coccinellid genus *Sasajiscymnus* Vandenberg. The analysis of our molecular data set clearly supported the genus *Sasajiscymnus* may be monophyletic but confirmation with an extended sampling is required. A fossil‐calibrated chronogram was generated by BEAST, indicating an origin of the genus at the end of the Cretaceous (77.87 Myr). Furthermore, a phylogenetic informativeness profile was generated to compare the phylogenetic properties of each gene more explicitly. The results showed that COI provides the strongest phylogenetic signal among all the genes, but Pepck, Topoisomerase, CADXM and CADMC are also relatively informative. Our results provide insight into the evolution of the genus *Sasajiscymnus*, and also enrich the molecular data resources for further study.

## Graphical abstract

We optimized five NPC fragments widely used in beetle phylogenetics by reducing the degenerate site of primers and the length of target genes slightly. These five NPC fragments and 6 other molecular loci were amplified to test the monophyly of the coccinellid genus *Sasajiscymnus*. The analysis of our molecular data set clearly supported the genus *Sasajiscymnus* may be monophyletic but confirmation with an extended sampling is required. A fossil‐calibrated chronogram was generated by BEAST, indicating an origin of the genus at the end of the Cretaceous (77.87 Myr). Furthermore, a phylogenetic informativeness profile was generated to compare the phylogenetic properties of each gene more explicitly. The results showed that COI provides the strongest phylogenetic signal among all the genes, but Pepck, Topoisomerase, CADXM, and CADMC are also relatively informative

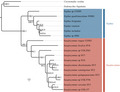

## 1 INTRODUCTION

Selecting appropriate molecular markers is crucial for inferring phylogenetic relationships and evolutionary history at different taxonomic levels. From a perspective of sequence data acquisition, mitochondrial genes and rDNA genes can be easily handled using universal primers and standard PCR (Caravas & Friedrich, 2013; Lin & Danforth, 2004). However, base compositional heterogeneity and among‐site rate variation of mitochondrial genes and alignment problems and slow rates of evolution in the case of rDNA genes can have a negative impact on phylogenetic tree inference (Bernt et al., 2013; Cameron, 2014). Nuclear protein‐coding (NPC) markers possess features that make them suitable for phylogenetic reconstruction. They are less prone to biased base composition than mitochondrial genes. Due to their distinct variation in the evolutionary rate (Lin & Danforth, 2004; Winkler et al., 2015), they have broad phylogenetic utility (Evangelista et al., 2019; Meusemann et al., 2010; Peters et al., 2017; Wipfler et al., 2019). A potential problem with NPC markers is that they are often more difficult to amplify than traditional markers via standard PCR. Usually, nested PCR and Hemi‐nested PCR amplification strategies are employed to obtain NPC genes by using degenerate primers (Shen, Liang, & Zhang, 2012; Shen, Liang, Feng, Chen, & Zhang, 2013; Wild & Maddison, 2008). Nonetheless, the amplification success rate of degenerate primers is low, and they tend to amplify nontargeted regions (Lin & Danforth, 2004; Pistone, Mugu, & Jordal, 2016). Although primer development and amplification across taxa for NPC markers can be relatively difficult compared to mitochondrial and rDNA markers, some researchers argued that improved primer design may be helpful to obtain targeted regions and sequencing regularity at appreciable levels (Pistone et al., 2016; Winkler et al., 2015).

The genus *Sasajiscymnus* Vandenberg belongs to the tribe Scymnini in the family Coccinellidae. The species of this genus are small and can be recognized by 9 antennomeres, a carinate prosternal process, a 1st abdominal ventrite with incomplete postcoxal lines, and 3 tarsomeres tarsi (Chapin, 1962; Sasaji, 1971; Vandenberg, 2004). They are specialized predators of hemipteran pests such as aphids, mealybugs, and scale insects (Conway, Culin, Burgess, & Allard, 2010; Pang & Gordon, 1986). Up to the present, sixty‐three species of this genus were described from Asia, Africa, and Oceania (Sasaji & McClure, 1997). Only a few species of *Sasajiscymnus* have been included in phylogenetic studies of Coccinellidae so far. Based on results of phylogenetic analyses of a combination of molecular and morphological data, Seago, Giorgi, Li, and Ślipiński (2011) proposed an affinity between *Sasajiscymnus* and *Nephus*, but with limited support. Subsequently, Robertson et al. (2015) presented a phylogeny of Cucujoidea based on molecular data and including 87 coccinellid terminals. They recovered *Sasajiscymnus* and *Scymnus* + *Nephus* formed a clade also with limited support. Che et al. (2017), in a phylogenomic study based on 95 NPC genes, recovered closely related between *Sasajiscymnus* and *Axinoscymnus* + *Nephus*. Due to only a single species of *Sasajiscymnus* included in the above‐mentioned analyses, the monophyly of the genus has not been tested.

In this study, we optimized five NPC fragments widely used in beetle phylogenetics by reducing the degenerate site of primers and the length of target genes slightly (Escalona et al., 2017; Wild & Maddison, 2008). These five fragments combined with three mitochondrial genes (COI, 12S, 16S) and three nuclear genes (18S, 28S, H3) were amplified by polymerase chain reaction (PCR) to address the phylogenetic relationships of *Sasajiscymnus*, using maximum likelihood and Bayesian inference. We also generated a fossil‐calibrated time tree to assess the time of origin and the pattern of diversification. A phylogenetic informativeness profile was also generated to evaluate the phylogenetic properties of the individual genes.

## 2 MATERIAL AND METHODS

### 2.1 Sampling

The study material was obtained from the Insect Collection, Department of Entomology, South China Agricultural University (SCAU). Specimens were collected from China by sweeping net in 2010. The samples were either stored in 95% ethanol or dried and pinned. Eight species of *Sasajiscymnus* were newly sequenced. Two additional species included in previous studies (Escalona et al., 2017; Robertson et al., 2015) were added as in‐group taxa. Six species of *Nephus* (sequences were newly generated for two species and obtained from GenBank for four others) were used as outgroup as possible sister group (Seago et al., 2011) or at least closely related (Che et al., 2017; Robertson et al., 2015) of the *Sasajiscymuns*. Two species of Endomychidae were included as distant outgroup taxa to root the trees. A total of 19 species were included in the analyses (Table S1).

### 2.2 DNA extraction, PCR, and sequencing

Total genomic DNA was extracted using a DNeasy Blood and Tissue kit (Qiagen, China) and following the manufacturer's protocol. The dried specimens were pretreated with 0.9% NaCl buffer before DNA extraction according to the previous study (Huang, Xie, Liang, Wang, & Chen, 2019). Thirteen gene fragments were sequenced: mitochondrial cytochrome‐c oxidase subunit I (COI), cytochrome‐c oxidase subunit II (COII), cytochrome‐b (Cytb), 12S rRNA (12S) and 16S rRNA (16S), and nuclear 18S rRNA (18S), 28S rRNA (28S), histone subunit (H3), two parts of carbamoyl‐phosphate synthetase (CADXM and CADMC), Topoisomerase (TOPO), Wingless (WGL) and Pepck (PE). The primers are listed in Table S2. For the PCR, we followed protocols by Wild and Maddison (2008), Robertson et al. (2013), and Escalona et al. (2017). Product yield, specificity, and potential contamination were monitored by agarose gel electrophoresis. DNA fragments were sequenced in both directions with sufficient overlap to ensure the accuracy of sequence data.

### 2.3 Primer design and sequence alignment

**FIGURE 1:**
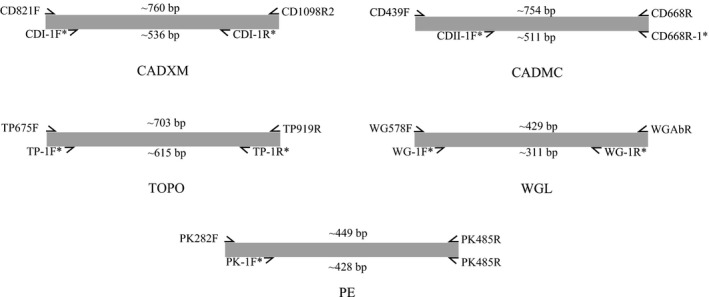
Schematic representation of amplification region of newly designed primers for five nuclear protein‐coding fragments. CADXM and CADMC: two parts of carbamoyl‐phosphate synthetase; TOPO: Topoisomerase; WGL: Wingless; PE: Pepck. The asterisk in the upper right corner of the primer name indicates that the primer was newly generated in this study

We used the program Primer 3 (Rozen & Skaletsky, 2000) in Geneious 7.1.4 (Kearse et al., 2012) to optimize the primers for CADXM, CADMC, TOPO, WGL, and PE (Figure 1). The sequences of each fragment of different coccinellid taxa were downloaded from GenBank as templates (Table S3). For each primer pair, the following parameters were set: The oligomer ranged from 18 bp to 28 bp, GC content ranged from 30% to 70% and the Tm value ranged from 50 to 65.

All sequences were initially assembled to contigs and examined using Geneious (Kearse et al., 2012). Sequences of each protein‐coding gene were aligned individually based on codon‐based multiple alignments using the MAFFT algorithm implemented in Geneious. Sequences of each RNA gene were individually aligned using MAFFT 7.0 with G‐INS‐I strategy (Katoh & Standley, 2013) and ambiguously aligned sites were removed using GBlock v0.91b with default settings (Talavera & Castresana, 2007). Afterward, all gene fragments were combined using SequenceMatrix (Vaidya, Lohman, & Meier, 2011).

### 2.4 Phylogenetic analyses

We used maximum likelihood (ML) and Bayesian inference (BI) approaches to explore their phylogenetic relationship. Before the analyses, PartitionFinder 2.1.1 (Lanfear, Frandsen, Wright, Senfeld, & Calcott, 2016) was used to determine the best‐fit partition scheme and corresponding models of nucleotide substitution for the concatenated data with the greedy algorithm and corrected by Akaike Information Criterion (AICc). ML analysis was conducted in RAxML 8.2.8 (Stamatakis, 2014) with the 1,000 rapid bootstrapping replicates on the CIPRES Science Gateway (Miller, Pfeiffer, & Schwartz, 2010). BI analysis was implemented in MrBayes 3.2.6 (Ronquist & Huelsenbeck, 2003) with four chains (three heated chains and one cold chain) running for 30 million generations and sampling every 10,000 generations. We discard the first 25% of trees as “burn‐in” and the remaining trees were used to generate a majority‐rule consensus tree. The analysis was carried out on the CIPRES Science Gateway.

### 2.5 Divergence time estimation

Divergence times of the *Sasajiscymnus* were estimated with BEAST 1.8.4 (Drummond, Suchard, Xie, & Rambaut, 2012) based on the concatenated sequence data through the CIPRES Science Gateway. All partitions were treated as unlinked for substitution models but as linked for clock models and trees. An amber fossil of the genus *Nephus* (*Nephus subcircularis*) from the Lowermost Eocene was used as a calibration node (Kirejtshuk & Nel, 2012). We conservatively assigned a minimum age of 40 Myr to the crown node of *Nephus*. The prior estimate of divergence date was specified using lognormal distributions, with a mean of 30 and log (stdev) of 0.75, which is consistent with the dating analyses of Coleoptera according to McKenna et al. (2015) and Toussaint et al. (2017). We performed analyses using the Yule branching process prior, assuming an uncorrelated lognormal distribution clock model. The Markov Chain Monte Carlo (MCMC) analysis was conducted using two independent runs of 50 million generations sampled every 10,000 generations. Tracer 1.6 was used to examine the trace plots and effective sample size (ESS) value to ensure that each parameter had been appropriately sampled (ESS > 200). We used the program Treeannotator v1.8.4 (Drummond et al., 2012) to obtain the maximum clade credibility, after removing the first 25% of topologies as “burn‐in” timetree for viewing in the program Figtree v1.4.0 (Rambaut, 2009).

### 2.6 Phylogenetic informativeness estimation

We used phylogenetic informativeness (PI) profiles to quantify the relative contribution of each partition to the resulted tree as implemented in PhyDesign (http://phydesign.townsend.yale.edu/) (López‐Giráldez & Townsend, 2011). An ultrametric tree file and an alignment were required for estimating phylogenetic informativeness. The input ultrametric tree was obtained from the above‐mentioned divergence time analysis. The peak of the PI distribution is suggested to predict the maximum phylogenetic informativeness for the corresponding partition. We used the concatenated DNA alignment as the input alignment with each gene as a partition.

## 3 RESULTS

### 3.1 Sequences alignment, model selection, and phylogenetic reconstruction

PartitionFinder identified 8 partitions for the combined dataset and the corresponding substitution model for each partition are shown in Table 1. Due to the currently impossible to specify different models of substitution for different partitions in RAxML, we used the partitions selected in PartitionFinder with a GTR + I + G model. For BI analysis, each partition and corresponding parameters used in BI analysis are summarized in Table 1.

**TABLE 1:** The best‐fit partitioning schemes and corresponding partition models used in BI analysis

Partitioned dataset	Nucleotide model	Implemented parameters in BI analysis
12S + 16S	GTR + I + G	nst = 6 rates = invgamma
18S	GTR + I + G	nst = 6 rates = invgamma
28S	GTR + I + G	nst = 6 rates = invgamma
CADXM	TRN + I + G	nst = 6 rates = invgamma
CADMC + PE	GTR + I + G	nst = 6 rates = invgamma
COI	GTR + I + G	nst = 6 rates = invgamma
H3	HKY + I + G	nst = 2 rates = invgamma
TOPO + WGL	GTR + I + G	nst = 6 rates = invgamma

**FIGURE 2:**
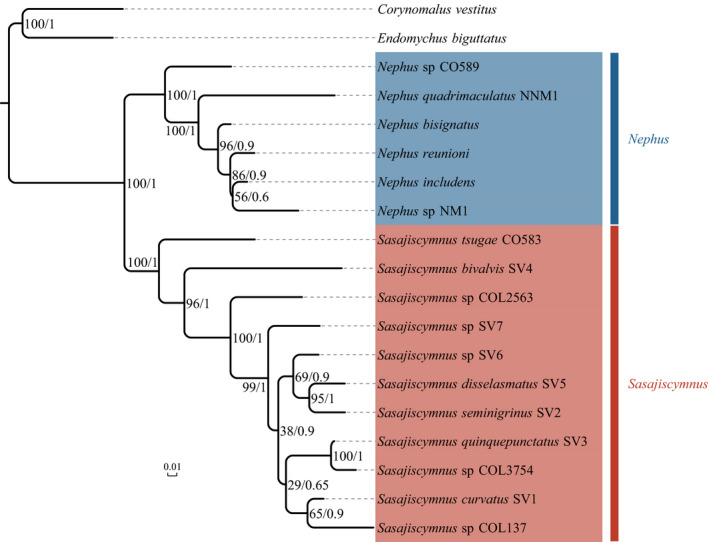
Phylogeny of *Sasajiscymnus* and related outgroups based on 11 molecular fragments using RAxML and MrBayes. Branch lengths are proportional to the number of substitutions per nucleotide position (scale bar in nucleotide substitutions per site). Numbers at nodes represent bootstrap value and posterior probability, respectively

Topologies retrieved by ML and BI methods based on the combined dataset were congruent, and all yielded a well‐supported and highly resolved phylogeny with high statistical support values for a large number of nodes (Figure 2). All specimens of *Sasajiscymnus* or *Nephus* respectively formed a clade with strong support values in both ML and BI analyses. Within *Sasajiscymnus*, *S. tsugae* CO583 was placed as sister to all other species of the genus. The following relationships were also recovered with high support: (*Sasajiscymnus* sp. COL3754 + *S. quinquepunctatus* SV3) + (*S. seminigrinus* SV2 + *S*. *disselasmatus* SV5). Within *Nephus*, *Nephus* sp. CO589 was the sister group of the rest of the genus.

### 3.2 Estimation of divergence time and phylogenetic informativeness

**FIGURE 3:**
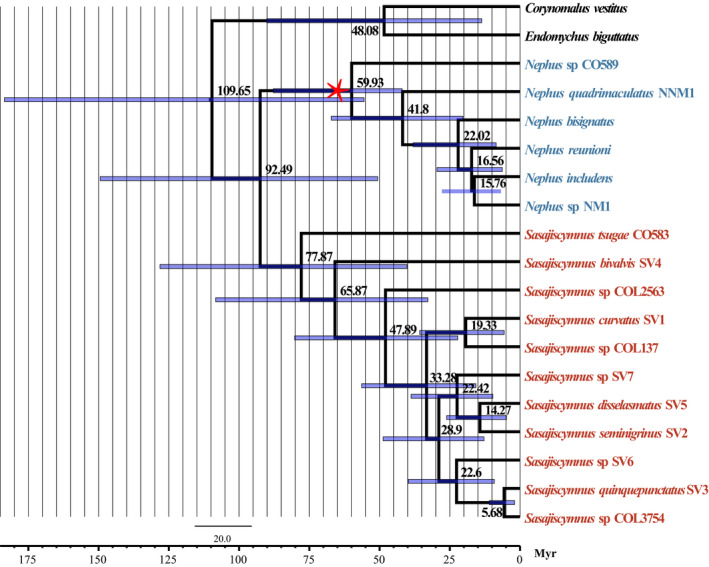
Chronogram depicting estimated divergence times of *Sasajiscymnus* based on 11 molecular fragments for analyses run with one fossil calibration (red asterisk). Nodes on the chronogram represent means of the probability distributions for node ages with time intervals for 95% probability of actual age represented as blue bars

The resulting consensus topology from the molecular clock calculation was largely identical with the Bayesian consensus tree from MrBayes and the ML tree obtained with RAxML (Figure 3). Our fossil‐calibrated Chronogram suggests a Late Cretaceous origin of *Sasajiscymnus* at 77.87 Myr and a Late Paleocene origin of *Nephus* at 59.93 Myr. Within *Sasajiscymnus*, *S. tsugae* CO583 diverged early from the base of *Sasasjiscymnus* at 77.87 Myr, and *S. bivalvis* SV4 diverged from the rest of *Sasajiscymnus* 12 Myr later, at 65.87 Myr. Diversification within the genus was shown to begin during the Early Oligocene (33.28 Myr).

**FIGURE 4:**
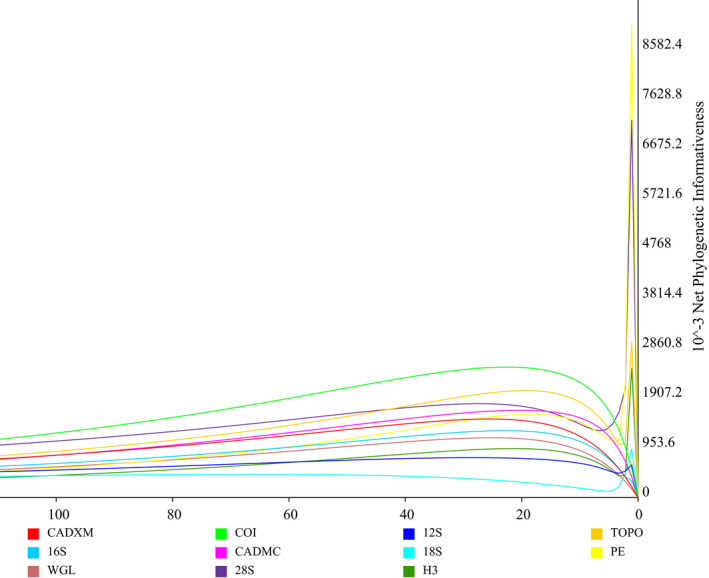
Net phylogenetic informativeness curves for each fragment of the sequence data in relation to time

In order to assess the relative contributions of the individual genes to the phylogeny, the PI profiles are plotted overtime against the chronogram (Figure 4). Phylogenetic informativeness profiles with relatively low and gradual peaks can be seen in 18S and protein‐coding genes, including COI, TOPO, and CADMC. Regarding rRNA genes, the highest net PI is obtained for 28S, followed by 16S and 12S, while the slow‐evolving 18S gene contains comparatively little information. However, a peak of the PI can be recognized from PE, 28S, TOPO, H3, and 18S near the terminal branches. Overall, COI provides the most phylogenetic signal among all the genes.

## 4 DISCUSSION

Our optimization of the five NPC fragments yielded a reduced length of CADXM, CADMC, PE, TOPO, and WGL from the original 760 bp, 754 bp, 449 bp, 703 bp and 429 bp to 536 bp, 511 bp, 428 bp, 615 bp and 311 bp, respectively. These five NPC fragments were developed and evaluated by Wild and Maddison (2008) in a phylogenetic analysis of beetles, and they were applied to process the phylogenetic inference of Coccinellidae on the genus level (Escalona et al., 2017). In our PCR‐based experiment, the amplification success rate of these genes was poor before optimization, especially for dry pinned specimens (data not shown). The novel primers produced a discrete band of the expected size and showed a good performance for these five NPC fragments. The redesigned primers can distinctly facilitate the acquisition of molecular data in phylogenetic studies of other ladybird beetle lineages when using these NPC fragments.

Although our enhanced test of the monophyly of *Sasajiscymnus* with six added “traditional genes” clearly provided robust support for the status of this genus as a clade, we cannot draw a conclusion that this genus was monophyletic due to our limited taxon sampling from other distribution regions and the type species has not been included in this study*. Nephus* was also confirmed as a monophyletic group, as in a previous study based on COI, 12S, 16S, 18S, and 28S (Magro, Lecompte, Magné, Hemptinne, & Crouau‐Roy, 2010). An affinity between *Sasajiscymnus* and *Nephus* was revealed by simultaneous analyses of molecular and morphological data, but with limited support (Seago et al., 2011). Nevertheless, other studies recovered the genus *Sasasjicymnus* either as sister group of *Scymnus* + *Nephus* (Robertson et al., 2015) or of *Axinoscymnus* + *Nephus* (Che et al., 2017). Due to the lack of species of *Axinoscymnus* and S*cymnus* in our sampling, we cannot draw a conclusion on the sister group of *Sasajiscymnus*. However, a very close relationship of *Nephus* with *Sasajiscymnus* is very likely. Further study with an extended sampling should address the genus‐level relationships within Scymnini. Moreover, a comprehensive evaluation of morphological features in the tribe is still wanting.

Our fossil‐calibrated chronogram generated by BEAST suggests the age of origin of *Sasajiscymnus* around the end of Cretaceous (77.87 Myr), and an origin of *Nephus* in the Late Paleocene around 59.93 Myr. The diversification of *Sasajiscymnus* occurred *ca*. 33.28 Myr in the Oligocene, between the Priabonian (37.8–33.9 Myr) and Rupelian (33.9–27.82 Myr). However, a considerably wider range of origin time of *Sasajiscymnus* (95% HPD: 40.24 Myr–128.16 Myr) and other nodes within the branch is conceivable based on the results of these analyses. It is acknowledged that limited gene sampling can cause wider confidence interval and tends to produce random errors relative to more extensive sampling of genes in divergence time estimations under Bayesian relaxed clock methods (Mulcahy et al., 2012; Shen et al., 2015). Battistuzzi, Filipski, Hedges, and Kumar (2010) also pointed out that analyzing multiple loci can improve the precision of posterior time estimation using this approach. Therefore, future studies should increase the number of specimens and genes to improve the robustness of divergence time estimation of *Sasajiscymnus*, to elucidate evolutionary processes in this genus and related groups, including biogeographic patterns, species diversification, and phenotypic evolution.

Quantifying the relative contribution of the genes by using phylogenetic informativeness profiles is important for selecting appropriate molecular markers to conduct phylogenetic analyses on different taxonomic levels. Our analysis indicates that COI provides the strongest phylogenetic signal for the addressed issue (Figure 4). In previous studies on the entire Coccinellidae, COI was commonly used and confirmed as informative (Escalona et al., 2017; Magro et al., 2010; McKenna et al., 2015; Robertson et al., 2015; Seago et al., 2011). Our results showed that the NPC fragments (except for WGL) are relatively informative and thus contribute to the reconstruction of the genus‐level relationships. Moreover, it is likely that the performance would be increased if these sequence data could be obtained for each included taxon. Among four rRNA genes, 28S rRNA was most informative, followed by 16S and 12S, and finally 18S rRNA. This result is in agreement with Yang and Zhang (2015), who showed that 28S rRNA was very informative in phylogenetic analyses of Nymphalidae (Lepidoptera). Based on these results, we suggested that widely used NPC genes and “traditional markers” such as COI and 28S rRNA can be combined in future phylogenetic analyses of Coccinellidae.

## 5 CONCLUSIONS

We propose the genus *Sasajiscymnus* may be monophyletic but confirmation with an extended outgroup sampling is required. A fossil‐calibrated chronogram suggests an origin of the genus around the end of the Cretaceous (77.87 Myr). Our phylogenetic informativeness profiles showed that COI provides the strongest phylogenetic signal, but the NPC fragments PE, TOPO, CADXM, and CADMC are also relatively informative. Increased sampling of species of areas highlighted as potential hotspots, as well as across all biogeographic regions could provide further insight in the evolution of *Sasajiscymnus* and related groups. We recommend that COI and 28S rRNA should be combined with NPC genes in reconstructing the phylogenetic analyses of Coccinellidae in future studies. The phenotypic character evolution (immature stages and adults) should be addressed based on a robust molecular phylogeny of the family.

## CONFLICT OF INTEREST

None declared.

## AUTHOR CONTRIBUTIONS

**Weidong Huang:** Conceptualization (equal); formal analysis (equal); writing–original draft (equal). **Xiufeng Xie:** Formal analysis (supporting); investigation (supporting); methodology (supporting). **Feng Peng:** Formal analysis (supporting); investigation (supporting). **Xinyue Liang:** Formal analysis (supporting); investigation (supporting). **Xingmin Wang:** Conceptualization (supporting); resources (equal); supervision (supporting); writing–original draft (supporting). **Xiaosheng Chen:** Conceptualization (lead); data curation (lead); funding acquisition (lead); investigation (lead); project administration (lead); supervision (lead); writing–original draft (lead).

## Supporting information

Table S1

Table S2

Table S3

## Data Availability

The DNA sequences reported in this study have been deposited in GenBank under accession number: MT096438‐MT096511.
