# Supplementary material for: Optimizing the widely used nuclear protein‐coding gene primers in beetle phylogenies and their application in the genus Sasajiscymnus Vandenberg (Coleoptera: Coccinellidae)
Source: Ecol Evol. 2020 Jun 28;10(14):7731–8. doi: 10.1002/ece3.6497 (PMC7391345; doi:10.1002/ece3.6497)
Supplement: Supplementary file 2 — Table S2 [file ECE3-10-7731-s002.docx]

Table S2 Primers and corresponding information for genes used in this study

| **Gene** | **Primer** | **Sequences (5’-3’)** | **References** |
| --- | --- | --- | --- |
| CADXM | CD821F | AGCACGAAAATHGGNAGYTCNATGAARAG | Wild and Maddison, 2008 |
|  | CD1098R2 | GCTATGTTGTTNGGNAGYTGDCCNCCCAT | Wild and Maddison, 2008 |
|  | CDI-1F | ATGGNATWGGHRGVAAATTCGARGAAGC | This study |
|  | CDI-1R | AGTTCRCKVAGRCANCCAACDGCACACC | This study |
| CADMC | CD439F | TTCAGTGTACARTTYCAYCCHGARCAYAC | Wild and Maddison, 2008 |
|  | CD668R | ACGACTTCATAYTCNACYTCYTTCCA | Wild and Maddison, 2008 |
|  | CDII-1F | CAAGCRATHAARGCHTTRVGNGAAGA | This study |
|  | CD668R-1 | ACGACTTCATAYTCNACYTCTTTCCA | This study |
| Pepck | PK282F | GAAGGATGGCTBGCNGARCAYATG | Wild and Maddison, 2008 |
|  | PK485R | GCAGCVGTNGCYTCRCTYCTCAT | Wild and Maddison, 2008 |
|  | PK-1F | ARGATATRGCNTGGATGAGATTCG | This study |
| TOPO | TP675F | GAGGACCAAGCNGAYACNGTDGGTTGTTG | Wild and Maddison, 2008 |
|  | TP919R | GTCTCTTTGCGTYTTRTTRTADATYTTYTC | Wild and Maddison, 2008 |
|  | TP-1F | TCGTTDCGWGTHGARCACATHGA | This study |
|  | TP-1R | AAGCRATHGYTTTRTTYTCRTCR | This study |
| WGL | WG578F | TGCACNGTGAARACYTGCTGGATG | Wild and Maddison, 2008 |
|  | WGAbR | ACYTCGCAGCACCARTGGAA | Wild and Maddison, 2008 |
|  | WG-1F | TTATCGGAGACATCCTGAARGA | This study |
|  | WG-1R | TTCCGCAGCACATRATRTCRC | This study |
| 18S | 18S a0.7 | ATTAAAGTTGTTGCGGTT | Robertson *et al.*, 2013 |
|  | 18S bi | GAGTCTCGTTCGTTATCGGA | Robertson *et al*., 2013 |
|  | 18S a2.0 | ATGGTTGCAAAGCTGAAAC | Robertson *et al*., 2013 |
|  | 18S 9r | GATCCTTCCGCAGGTTCACCTAC | Robertson *et al*., 2013 |
| 28S | 28S A | GACCCGTCTTGAAGCACG | Robertson *et al*., 2013 |
|  | 28S Rd 5b | CCACAGCGCCAGTTCTGCTTAC | Robertson *et al*., 2013 |
|  | 28S Rd 4.8a | ACCTATTCTCAAACTTTAAATGG | Robertson *et al*., 2013 |
|  | 28S Rd 7b1 | GACTTCCCTTACCTACAT | Robertson *et al*., 2013 |
| H3 | H3 AF | ATGGCTCGTACCAAGCAGACVGC | Robertson et al., 2013 |
|  | H3 AR | ATATCCTTRGGCATRATRGTGAC | Robertson et al., 2013 |
| COI | Jerry | CAACATTTATTTTGATTTTTT | Timmermans *et al*., 2010 |
|  | Spat | GCACTAWTCTGCCATATTAGA | Timmermans *et al*., 2010 |
| 12S | 12S ai | AAACTACGATTAGATACCCTATTAT | Robertson *et al*., 2013 |
|  | 12S bi | AAGAGCGACGGGCGATGTGT | Robertson *et al*., 2013 |
| 16S | 16S A | CGCCTGTTTATCAAAAACAT | Robertson *et al*., 2013 |
|  | 16S B | CTCCGGTTTGAACTCAGATCA | Robertson *et al*., 2013 |
